# Trends in First-Line Antiretroviral Therapy in Asia: Results from the TREAT Asia HIV Observational Database

**DOI:** 10.1371/journal.pone.0106525

**Published:** 2014-09-03

**Authors:** David Charles Boettiger, Stephen Kerr, Rossana Ditangco, Tuti Parwati Merati, Thuy Thi Thanh Pham, Romanee Chaiwarith, Sasisopin Kiertiburanakul, Chung Ki Patrick Li, Nagalingeswaran Kumarasamy, Saphonn Vonthanak, Christopher Lee, Nguyen Van Kinh, Sanjay Pujari, Wing Wai Wong, Adeeba Kamarulzaman, Fujie Zhang, Evy Yunihastuti, Jun Yong Choi, Shinichi Oka, Oon Tek Ng, Pacharee Kantipong, Mahiran Mustafa, Winai Ratanasuwan, Annette Sohn, Matthew Law

**Affiliations:** 1 The Kirby Institute, UNSW Australia, Sydney, New South Wales, Australia; 2 HIV-NAT, Thai Red Cross AIDS Research Centre, Bangkok, Thailand; 3 Research Institute for Tropical Medicine, Manila, Philippines; 4 Udayana University, Sanglah Hospital, Denpesar, Indonesia; 5 Bach Mai Hospital, Hanoi, Vietnam; 6 Research Institute for Health Sciences, Chiang Mai, Thailand; 7 Faculty of Medicine Ramathibodi Hospital, Mahidol University, Bangkok, Thailand; 8 Queen Elizabeth Hospital, Hong Kong, China SAR; 9 YRG Centre for AIDS Research and Education, Chennai, India; 10 National Center for HIV/AIDS, Dermatology & STDs, Phnom Penh, Cambodia; 11 Hospital Sungai Buloh, Sungai Buloh, Malaysia; 12 National Hospital of Tropical Diseases, Hanoi, Vietnam; 13 Institute of Infectious Diseases, Pune, India; 14 Taipei Veterans General Hospital, Taipei, Taiwan; 15 University of Malaya Medical Centre, Kuala Lumpur, Malaysia; 16 Beijing Ditan Hospital, Capital Medical University, Beijing, China; 17 Working Group on AIDS Faculty of Medicine, University of Indonesia/Cipto Mangunkusumo Hospital, Jakarta, Indonesia; 18 Division of Infectious Diseases, Department of Internal Medicine, Yonsei University College of Medicine, Seoul, South Korea; 19 National Center for Global Health and Medicine, Tokyo, Japan; 20 Tan Tock Seng Hospital, Singapore, Singapore; 21 Chiang Rai Prachanukroh Hospital, Chiang Rai, Thailand; 22 Hospital Raja Perempuan Zainab II, Kota Bharu, Malaysia; 23 Faculty of Medicine, Siriraj Hospital, Mahidol University, Bangkok, Thailand; 24 TREAT Asia, amfAR – The Foundation for AIDS Research, Bangkok, Thailand; Infectious Disease Service, United States of America

## Abstract

**Background:**

Antiretroviral therapy (ART) has evolved rapidly since its beginnings. This analysis describes trends in first-line ART use in Asia and their impact on treatment outcomes.

**Methods:**

Patients in the TREAT Asia HIV Observational Database receiving first-line ART for ≥6 months were included. Predictors of treatment failure and treatment modification were assessed.

**Results:**

Data from 4662 eligible patients was analysed. Patients started ART in 2003–2006 (n = 1419), 2007–2010 (n = 2690) and 2011–2013 (n = 553). During the observation period, tenofovir, zidovudine and abacavir use largely replaced stavudine. Stavudine was prescribed to 5.8% of ART starters in 2012/13. Efavirenz use increased at the expense of nevirapine, although both continue to be used extensively (47.5% and 34.5% of patients in 2012/13, respectively). Protease inhibitor use dropped after 2004. The rate of treatment failure or modification declined over time (22.1 [95%CI 20.7–23.5] events per 100 patient/years in 2003–2006, 15.8 [14.9–16.8] in 2007–2010, and 11.6 [9.4–14.2] in 2011–2013). Adjustment for ART regimen had little impact on the temporal decline in treatment failure rates but substantially attenuated the temporal decline in rates of modification due to adverse event. In the final multivariate model, treatment modification due to adverse event was significantly predicted by earlier period of ART initiation (hazard ratio 0.52 [95%CI 0.33–0.81], p = 0.004 for 2011–2013 versus 2003–2006), older age (1.56 [1.19–2.04], p = 0.001 for ≥50 years versus <30years), female sex (1.29 [1.11–1.50], p = 0.001 versus male), positive hepatitis C status (1.33 [1.06–1.66], p = 0.013 versus negative), and ART regimen (11.36 [6.28–20.54], p<0.001 for stavudine-based regimens versus tenofovir-based).

**Conclusions:**

The observed trends in first-line ART use in Asia reflect changes in drug availability, global treatment recommendations and prescriber preferences over the past decade. These changes have contributed to a declining rate of treatment modification due to adverse event, but not to reductions in treatment failure.

## Introduction

The 2013 World Health Organization (WHO) guidelines recommend that first-line antiretroviral therapy (ART) optimally consist of the non-nucleoside reverse transcriptase inhibitor (NNRTI), efavirenz (EFV), and two nucleoside reverse transcriptase inhibitors (NRTIs), lamivudine (3TC)/emtricitabine (FTC) and tenofovir (TDF).[1] US and UK guidelines state that an NNRTI, protease inhibitor (PI) or a newer class antiretroviral can be used to support the NRTI backbone.[2], [3] Currently, most Asian clinics only have sufficient resources to comply with earlier, more generalised guidelines which recommended a dual NRTI + NNRTI first-line regimen.[4], [5] PIs and newer classes of antiretrovirals remain expensive first-line options, however, dual NRTI + PI therapy is the most common second-line alternative used in Asia.

3TC and FTC are structurally and functionally very similar and both exhibit excellent efficacy and safety. Either agent is an essential component of first-line ART. Since 2010, the WHO has strongly recommended against the use of stavudine (d4T) due to its serious long-term and potentially irreversible toxicities such as peripheral neuropathy and lipodystrophy.[4] TDF and zidovudine (AZT) are popular alternatives recommended by the WHO [1], [4], [5].

NNRTI preference is largely driven by local availability and patient tolerance. In terms of first-line efficacy, nevirapine (NVP) and EFV were long considered equivalent.[6] Importantly however, a recent trial found that the virological efficacy of NVP was inferior to that of EFV in HIV-tuberculosis co-infected patients.[7] Further, a systematic review by Shubber *et al* (2013) found that patients on NVP were more than twice as likely to discontinue treatment due to an adverse event compared to patients on EFV.[8] The 2013 WHO guidelines [1] state that ritonavir-boosted atazanavir (ATV/r) and lopinavir (LPV/r) are the preferred (second-line) PI options. Darunavir (DRV/r) is an alternative but is currently not available as a fixed-dose combination and is prohibitively expensive in lower-income countries.

Several studies have evaluated ART usage trends in populations outside of Asia.[9]–[12] This work reflects developments in knowledge and guidance on first-line ART. Further study has also demonstrated that expanded use of more potent ART over time precedes improved long-term survival in HIV-infected patients.[12] Knowledge of trends in ART usage in Asia and how these have impacted treatment outcomes is currently lacking.

The objective of this analysis is to summarize trends in first-line ART use over the past decade within an Asian cohort and investigate whether temporal changes in the rate of treatment failure and modification are attributable to changes in the use of ART.

## Methods

The study population consisted of HIV-infected patients enrolled in the TREAT Asia HIV Observational Database (TAHOD) and/or the TREAT Asia Studies to Evaluate Resistance-Monitoring (TASER-M). These cohorts contribute to the International Epidemiologic Databases to Evaluate AIDS (IeDEA) global consortium and have been described previously.[13], [14] Briefly, TAHOD is an observational study of patients with HIV involving 21 adult treatment centers in 12 countries and territories of varying income levels in Asia, which aims to assess HIV disease natural history in treated and untreated patients in the region. Retrospective and prospective data is collected at each site. Recruitment started in September 2003. TASER-M was a multi-center, cohort study monitoring development of HIV drug resistance in patients taking ART. Patients eligible for first- or second-line ART initiation were enrolled sequentially. Data on previous antiretroviral use was collected retrospectively. Patient recruitment commenced in March 2007 and ceased in 2011. Follow-up data continues to be collected as TASER-M was merged with TAHOD in 2012. Currently, each TAHOD site has contributed data from 100–450 patients. Data is transferred to the data management center at the Kirby Institute, Sydney, Australia twice annually in March and September.

Ethics approval was granted for the TAHOD study design, methods and consent procedures by the University of New South Wales Human Research Ethics Committee. Site specific study governance was granted by site-relevant institutional review boards. Written informed consent was not sought in TAHOD unless required by a site’s local institutional review board. Informed consent was waived at some sites as information is collected via an anonymous case report form. All study procedures were developed in accordance with the revised 1975 Helsinki Declaration.

Patients from the September 2013 data transfer were included in this analysis if they started first-line ART in 2003 or later and had been on this regimen for ≥6 months. First-line ART was defined as the first regimen containing ≥3 antiretrovirals used for >14 days. Treatment breaks ≤14 days were ignored. Baseline was considered day one of first-line ART. Treatment failure was defined as the first occurrence of virological, immunological or clinical failure whilst on first-line ART, or a regimen change instigated due to treatment failure as indicated by the treating physician. Virological failure was considered a viral load >1,000copies/mL after 6 months of ART, confirmed within 6 months; immunological failure was defined as CD4 cell count <100 cells/mm^3^ or less than baseline after 6 months of ART, confirmed within 6 months and; clinical failure comprised of a new or recurrent WHO stage 3 or 4 illness or death after 6 months of ART. Treatment modification was defined as a change of ≥1 antiretroviral in the first-line regimen. Where multiple different outcomes occurred in a patient at the same time, treatment failure was given priority followed by modification due to adverse event, modification due to poor adherence, then modification due to other reasons.

A sensitivity analysis was performed to assess the impact of loss-to-follow-up by including this as an alternative outcome in our competing risk models. Patients in the main analysis with <12 months follow up time in TAHOD were excluded and lost-to-follow-up was defined as not being seen at the treating clinic for ≥12 months without documentation of transfer.

The window period for baseline CD4 cell count was within 3 months of first-line ART initiation. For baseline viral load it was up to 6 months before first-line ART initiation. The measurement taken closest to first-line ART initiation was used. Patients were considered hepatitis B co-infected if they had any record of a positive hepatitis B surface antigen test in the database and hepatitis C co-infected if they had any record of a positive hepatitis C antibody test.

### Statistical analysis

Predictors of treatment outcome were analyzed using Kaplan-Meier curves, cumulative incidence functions and competing risks regression adjusted by study site. Patients with missing data were included, but hazard ratios for missing categories are not reported except for ART adherence. Time-to-event was left censored. Right censoring occurred at the last recorded clinic visit whilst still on first-line ART.

Predictors to be considered in the multivariate model were selected based on a significance level of ≤0.15 in the univariate analysis. Predictors were retained in the multivariate model if one or more categories exhibited a p-value≤0.05.

Stata software version 12.1 was used for all statistical analysis.

## Results

A total of 4662 patients were eligible for inclusion in this analysis. Baseline data is presented in Table 1. Years of ART initiation were 2003 (n = 443), 2004 (n = 352), 2005 (n = 362), 2006 (n = 262), 2007 (n = 407), 2008 (n = 712), 2009 (n = 737), 2010 (n = 834), 2011 (n = 414), 2012/13 (n = 139). The majority of patients were male (69.3%) and exposed to HIV via heterosexual contact (62.7%). Median age at first-line ART initiation was 35.2 [interquartile range (IQR) 29.9–41.7] years, median CD4 cell count was 134 [IQR 45–229] cells/mm^3^, and median HIV viral load was 93,800 [IQR 27,617–254,000] copies/mL. d4T + NRTI + NNRTI (d4T/NNRTI) was initiated by 1663 (35.7%) patients, AZT + NRTI + NNRTI (AZT/NNRTI) by 1728 (37.1%) patients, TDF + NRTI + NNRTI (TDF/NNRTI) by 495 (10.6%) patients, and dual NRTI + PI (PI-based) by 568 (12.2%) patients. Other regimens were comprised of abacavir (ABC) + NRTI + NNRTI (n = 122, 2.6%), didanosine + NRTI + NNRTI (n = 38, 0.8%), all NRTI (n = 27, 0.6%), and dual NRTI + raltegravir (n = 13, 0.3%).

**Table 1:** Baseline data (n = 4662).

**Sex**	
Male	3232 (69.3%)
Female	1430 (30.7%)
**Age (years)** Median(IQR) = 35.2 (29.9–41.7)	
<30	1184 (25.4%)
30–39	2054 (44.1%)
40–49	989 (21.2%)
≥50	435 (9.3%)
**HIV exposure**	
Heterosexual	2922 (62.7%)
Homosexual	953 (20.4%)
IDU	424 (9.1%)
Other	363 (7.8%)
**HBV status**	
Negative	3305 (70.9%)
Positive	367 (7.9%)
Missing	990 (21.2%)
**HCV status**	
Negative	2870 (61.6%)
Positive	552 (11.8%)
Missing	1240 (26.6%)
**Baseline CD4 (cells/mm^3^)** Median (IQR) = 134 (45–229)	
>350	256 (5.5%)
≤350	3771 (80.9%)
Missing	635 (13.6%)
**Viral load (copies/ml)** Median (IQR) = 93,800 (27,617–254,000)	
≤100,000	1343 (28.8%)
>100,000	1192 (25.6%)
Missing	2127 (45.6%)
**AIDS prior to ART initiation**	
None known	2909 (62.4%)
Yes	1753 (37.6%)
**Prior mono/dual therapy**	
None known	4356 (93.4%)
Yes	306 (6.6%)
**Initial ART regimen**	
d4T/NNRTI	1663 (35.7%)
AZT/NNRTI	1728 (37.1%)
TDF/NNRTI	495 (10.6%)
PI-based	568 (12.2%)
Other	208 (4.5%)
**Year of ART initiation**	
2003–2006	1419 (30.4%)
2007–2010	2690 (57.7%)
2010–2013	553 (11.9%)
**Adherence data available**	
Yes	3050 (65.4%)
No	1612 (34.6%)

Exposure category *Other* includes those exposed to blood products and unknown exposures. A *(d4T/AZT/TDF)/NNRTI* regimen is d4T/AZT/TDF + another NRTI + NNRTI. A *PI-based* regimen is a dual NRTI + PI regimen. *Other* regimen refers to all other ART regimens. IQR = interquartile range; IDU = intravenous drug use; HBV = hepatitis B; HCV = hepatitis C; ART = antiretroviral therapy; PI = protease inhibitor; NNRTI = non-nucleoside reverse transcriptase inhibitor; d4T = stavudine; AZT = zidovudine; TDF = tenofovir.

**Figure 1:**
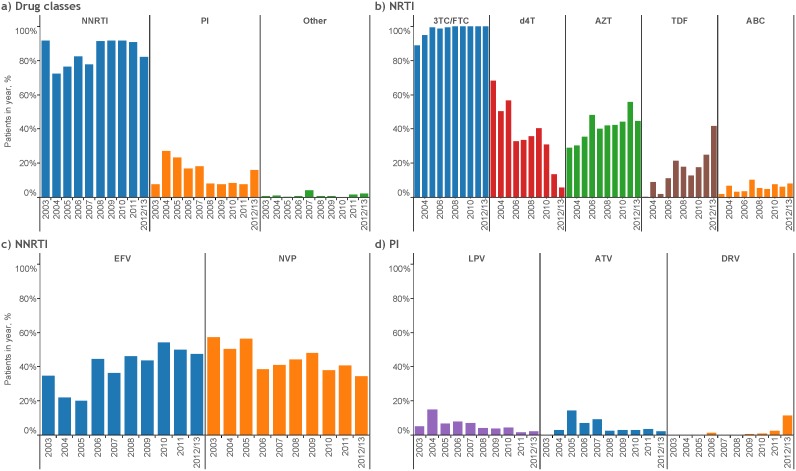
First-line ART use by year of initiation (n = 4662). a) Drug classes. NRTIs not represented as there was a single patient that initiated a regimen without an NRTI; b) NRTI. Not represented are didanosine (2.9% of patients overall) and zalcitabine (0.02%); c) NNRTI. Not represented is rilpivirine (0.13%); d) PI. Not represented are indinavir (0.66%), nelfinavir (0.39%), tipranavir (0.39%), saquinavir (0.17%), fosamprenavir (0.17%) and full-dose ritonavir (0.11%). ART = antiretroviral therapy; NRTI = nucleoside reverse transcriptase inhibitor; NNRTI = non-NRTI; PI = protease inhibitor; 3TC/FTC = lamivudine/emtricitabine; d4T = stavudine; AZT = zidovudine; TDF = tenofovir; ABC = abacavir; EFV = efavirenz; NVP = nevirapine; LPV = lopinavir; ATV = atazanavir; DRV = darunavir.

**Figure 2:**
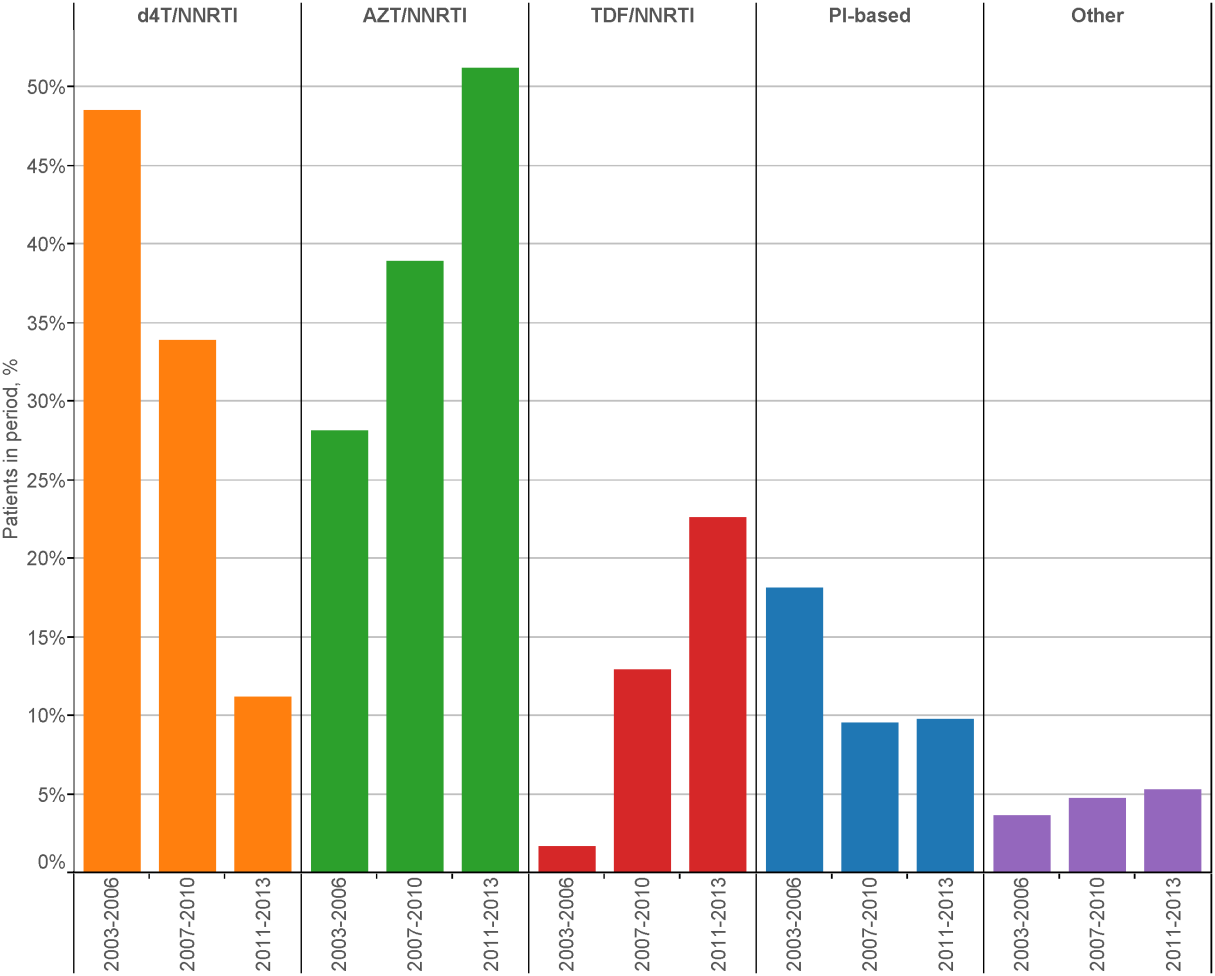
First-line regimen by period of initiation (n = 4662). A *(d4T/AZT/TDF)/NNRTI* regimen comprises d4T/AZT/TDF + another NRTI + NNRTI. A *PI-based* regimen comprises dual NRTI + PI. *Other* regimen refers to all other ART. d4T = stavudine; AZT = zidovudine; TDF = tenofovir; NNRTI = non-nucleoside reverse transcriptase inhibitor; NRTI = nucleoside reverse transcriptase inhibitor; PI = protease inhibitor.

Figure 1a shows that NNRTI use has been replacing PI use since 2004 although a small rise in PI use in 2012/13 is evident. Since 2005, 3TC/FTC has been used by almost 100% of ART initiators whilst TDF, AZT, and to a lesser extent, ABC, have been replacing the use of d4T (Figure 1b). Between 2003 and 2012/13, first-line d4T use dropped from 68.2% to 5.8%. Figure 1c shows that while EFV use increased steadily between 2003 and 2012/13 (from 34.8% to 47.5%), NVP use dropped (from 57.3% to 34.5%). LPV and ATV comprised the majority of PI use from 2003 to 2011 although 11.5% of patients in 2012/13 were using DRV compared with 2.2% for both LPV and ATV (Figure 1d). Figure 2 illustrates that in the periods 2003–2006, 2007–2010, and 2011–2013, d4T/NNRTI was used by 48.6%, 33.9% and 11.2% of patients, respectively. Over the same respective time periods, AZT/NNRTI use was 28.1%, 38.9% and 51.2%, TDF/NNRTI use was 1.6%, 12.9% and 22.6%, PI-based ART use was 18.1%, 9.6% and 9.8%, and the use of regimens other than those already defined was 3.6%, 4.8% and 5.2%.

Total follow-up time was 11,907 years. Median time on first-line ART was 2.0 (IQR 1.1–3.5) years. Treatment failure or modification occurred in 2121 (45.5%) patients at an incidence of 17.8 (95%CI 17.1–18.6) per 100 patient-years. Treatment failure occurred in 459 (9.8%) patients at an incidence of 3.9 (95%CI 3.5–4.2) per 100 patient-years. Fifty five treatment failures included documented virological failure (12.0%), 112 (24.4%) included documented immunological failure, 175 (38.1%) included documented clinical failure, and treatment modification with a recorded reason of failure occurred in 125 (27.2%) patients. The mortality rate was 0.5 per 100 patient-years (59 deaths in total). Treatment modification due to adverse event occurred in 815 (17.5%) patients at an incidence of 6.8 (95%CI 6.4–7.3) per 100 patient-years, and treatment modification due to poor adherence occurred in 26 (0.6%) patients at an incidence of 0.2 (95%CI 0.1–0.3) per 100 patient-years.

**Figure 3:**
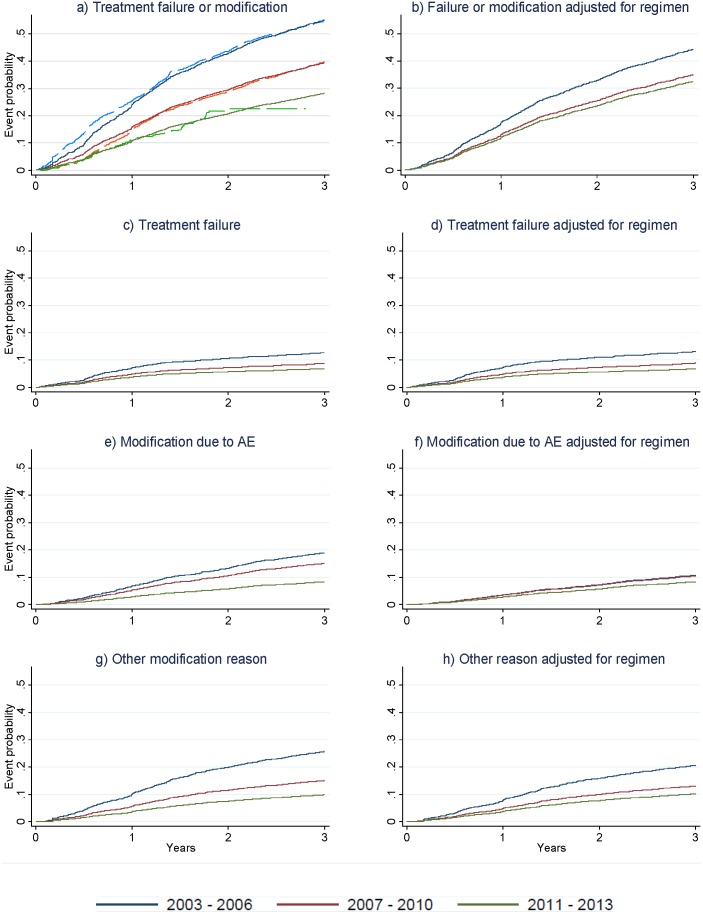
Cumulative probabilities of first-line ART failure or modification (n = 4662). Dashed lines in a) represent Kaplan-Meier curves. Solid lines in a) to h) represent competing risk regression estimates. Regimen was categorized as d4T/AZT/TDF + another NRTI + NNRTI, dual NRTI + PI, and other. Follow up is truncated at 3 years. ART = antiretroviral therapy; AE = adverse event; d4T = stavudine; AZT = zidovudine; TDF = tenofovir; NRTI = nucleoside reverse transcriptase inhibitor; NNRTI = non-nucleoside reverse transcriptase inhibitor; PI = protease inhibitor.

The rates of treatment failure or modification for patients starting ART between 2003 and 2006, 2007 and 2010, and 2011 and 2013 were 22.1 (95%CI 20.7–23.5), 15.8 (14.9–16.8) and 11.6 (9.4–14.2) per 100 patient-years, respectively. In univariate models describing time to treatment modification or failure, failure alone, modification due to adverse event, and modification due to other causes (i.e., not due to treatment failure or adverse event), later period of ART initiation was consistently predictive of a longer time-to-event (Figure 3a, c, e, g). In the univariate model of time to treatment modification due to poor adherence, there was no difference between the different periods of ART initiation (overall p for linear trend = 0.642, overall p for heterogeneity = 0.888). When the model for treatment modification or failure was adjusted for first-line ART regimen, the association between period of ART initiation and time to event was partly attenuated (Figure 3b). This was largely due to tempering of the association between period of ART initiation and time to modification due to adverse event, as seen in Figure 3f. Adjustment for treatment regimen partly attenuated the temporal decline in the rate of modification due to other causes (Figure 3h) but had little impact on the relationship between period of ART initiation and time to treatment failure (Figure 3d).

In the final multivariate model for treatment failure (Table 2), later periods of ART initiation were significantly protective (hazard ratio 0.50 [95%CI 0.32–0.77], p = 0.002 for 2011–2013 and 0.67 [0.53–0.84], p<0.001 for 2007–2010 versus 2003–2006). Older age (1.70 [1.22–2.37], p = 0.002 for ≥50 years versus <30 years), positive hepatitis C status (1.43 [1.04–1.96], p = 0.028 versus negative) and a prior AIDS diagnosis (1.32 [1.08–1.61], p = 0.006 versus no prior AIDS) were significant predictors of treatment failure. No difference in time to treatment failure was observed between first-line ART regimens (overall p for heterogeneity = 0.549). In the final multivariate model for treatment modification due to adverse event (Table 2), significant co-variables were period of ART initiation (hazard ratio 0.52 [95%CI 0.33–0.81], p = 0.004 for 2011–2013 and 0.80 [95%CI 0.68–0.95], p = 0.010 for 2007–2010 versus 2003–2006), first-line ART regimen (11.36 [6.28–20.54], p<0.001 for d4T/NNRTI, 3.56 [1.86–6.84], p<0.001 for PI-based, and 2.64 [1.44–4.83], p = 0.002 for AZT/NNRTI versus TDF/NNRTI), older age (1.56 [1.19–2.04], p = 0.001 for ≥50 years versus <30 years), female sex (1.29 [1.11–1.50], p = 0.001 versus male) and positive hepatitis C status (1.33 [1.06–1.66], p = 0.013 versus negative). Baseline CD4 cell count, baseline viral load, mode of HIV exposure, prior mono/dual antiretroviral exposure, hepatitis B co-infection and poor ART adherence were not significantly predictive of treatment failure or treatment modification due to adverse event.

**Table 2:** Competing risk models of treatment failure and treatment modification due to adverse event (n = 4662).

	Treatment failure						Modification due to adverse event					
Co-variables	Univariate HR (95%CI)	p	p overall	Multivariate^¥^HR (95%CI)	p	p overall	UnivariateHR (95%CI)	p	p overall	Multivariate^¥^HR (95%CI)	p	p overall
**Period of ART initiation^¤◊^**												
2003–2006	1.00			1.00			1.00			1.00		
2007–2010	0.63 (0.50–0.79)	<0.001		0.67 (0.53–0.84)	**<0.001**		0.64 (0.54–0.75)	<0.001		0.80 (0.68–0.95)	**0.010**	
2011–2013	0.45 (0.30–0.70)	<0.001	<0.001^†^	0.50 (0.32–0.77)	**0.002**	**<0.001^†^**	0.30 (0.19–0.47)	<0.001	<0.001^†^	0.52 (0.33–0.81)	**0.004**	**0.001^†^**
**Initial ART regimen^¤◊^**												
d4T/NNRTI	1.64 (1.08–2.48)	0.019		1.25 (0.82–1.91)	0.300		13.27 (7.38–23.85)	<0.001		11.36 (6.28–20.54)	**<0.001**	
AZT/NNRTI	1.41 (0.94–2.10)	0.096		1.21 (0.80–1.83)	0.357		3.03 (1.66–5.51)	<0.001		2.64 (1.44–4.83)	**0.002**	
TDF/NNRTI	1.00			1.00			1.00			1.00		
PI-based	1.33 (0.76–2.33)	0.313		1.16 (0.67–2.03)	0.593		3.86 (2.01–7.43)	<0.001		3.56 (1.86–6.84)	**<0.001**	
Other	1.69 (0.94–3.04)	0.080	0.149^‡^	1.58 (0.88–2.83)	0.127	0.549^‡^	1.61 (0.74–3.51)	0.234	<0.001^‡^	1.62 (0.74–3.52)	0.225	**<0.001^‡^**
**Age (years)^¤◊^**												
<30	1.00			1.00			1.00			1.00		
30–39	1.23 (0.97–1.57)	0.087		1.19 (0.93–1.52)	0.167		1.16 (0.96–1.40)	0.123		1.12 (0.92–1.36)	0.252	
40–49	1.22 (0.91–1.62)	0.180		1.21 (0.91–1.63)	0.194		1.46 (1.19–1.80)	<0.001		1.56 (1.26–1.92)	**<0.001**	
≥50	1.68 (1.20–2.35)	0.002	0.008^†^	1.70 (1.22–2.37)	**0.002**	**<0.006^†^**	1.49 (1.14–1.94)	0.003	<0.001^†^	1.56 (1.19–2.04)	**0.001**	**<0.001^†^**
**HCV status^¤◊^**												
Negative	1.00			1.00			1.00			1.00		
Positive	1.48 (1.08–2.02)	0.014		1.43 (1.04–1.96)	**0.028**		1.25 (1.00–1.56)	0.049		1.33 (1.06–1.66)	**0.013**	
**AIDS prior to ART initiation^¤^**												
None known	1.00			1.00			1.00			1.00		
Yes	1.41 (1.16–1.72)	0.001		1.32 (1.08–1.61)	**0.006**		1.06 (0.92–1.23)	0.419		0.88 (0.76–1.02)	0.099	
**Sex^◊^**												
Male	1.00			1.00			1.00			1.00		
Female	0.77 (0.61–0.97)	0.024		0.84 (0.67–1.06)	0.144		1.23 (1.07–1.43)	0.005		1.29 (1.11–1.50)	**0.001**	
**HIV exposure**												
Heterosexual	1.00			1.00			1.00			1.00		
Homosexual	0.67 (0.49–0.92)	0.012		0.81 (0.59–1.12)	0.201		0.66 (0.51–0.85)	0.001		1.00 (0.75–1.33)	0.999	
IDU	1.16 (0.82–1.65)	0.404		0.90 (0.59–1.36)	0.604		1.37 (1.02–1.85)	0.039		1.34 (0.95–1.90)	0.099	
Other	0.84 (0.56–1.26)	0.406	0.059^‡^	0.85 (0.56–1.28)	0.437	0.578^‡^	0.87 (0.62–1.22)	0.420	0.001^‡^	0.96 (0.67–1.38)	0.841	0.413^‡^
**Prior mono/dual therapy**												
None known	1.00			1.00			1.00			1.00		
Yes	1.30 (0.94–1.79)	0.113		1.22 (0.88–1.69)	0.240		1.16 (0.88–1.54)	0.282		1.12 (0.85–1.49)	0.410	
**Baseline CD4 (cells/mm^3^)**												
>350	1.00			1.00			1.00			1.00		
≤350	0.89 (0.60–1.31)	0.551		0.81 (0.54–1.20)	0.286		1.11 (0.77–1.62)	0.58		0.91 (0.62–1.33)	0.618	
Adherence*												
≥95%	1.00			1.00			1.00			1.00		
<95%	1.50 (0.83–2.72)	0.178		1.56 (0.86–2.82)	0.146		0.92 (0.56–1.53)	0.753		0.92 (0.56–1.53)	0.750	
Unknown	4.76 (3.82–5.92)	<0.001		5.77 (4.49–7.42)	<0.001		2.86 (2.45–3.35)	<0.001		2.25 (1.87–2.71)	<0.001	

All models were adjusted for study site. Baseline CD4 cell count and adherence were not significant in univariate analysis for either outcome but are presented out of interest, as is the missing adherence category. Exposure category *Other* includes those exposed to blood products and unknown exposures. A *(d4T/AZT/TDF)/NNRTI* regimen comprises d4T/AZT/TDF + another NRTI + NNRTI. A *PI-based* regimen comprises dual NRTI + PI. *Other* regimen refers to all other ART. ^¤^Included in final treatment failure model; ^◊^Included in final modification due to adverse event model; ^¥^Adjusted for co-variables included in the final model; *Time updated; †p overall for linear trend; ‡p overall for heterogeneity; HR = hazard ratio; ART = antiretroviral therapy; IDU = intravenous drug use; HCV = hepatitis C virus; PI = protease inhibitor; NNRTI = non-nucleoside reverse transcriptase inhibitor; d4T = stavudine; AZT = zidovudine; TDF = tenofovir.

In the sensitivity analysis, rates of loss-to-follow-up for patients starting ART between 2003 and 2006, 2007 and 2010, and 2011 and 2013 were 2.3 (95%CI 1.9–2.8), 3.2 (2.8–3.7) and 0.9 (0.4–2.0) per 100 patient-years, respectively. As evidenced in Table S1, the final models and hazard ratios were very similar with and without adjustment for loss-to-follow-up.

## Discussion

This analysis describes the trends in first-line ART use in an Asian observational cohort over the past decade. TDF, AZT, and ABC have been steadily replacing the use of d4T. EFV has become increasingly popular at the expense of NVP, although both continue to be used extensively. PI use has dropped since 2004. The rate of first-line treatment failure declined over time and this relationship was unaffected by adjustment for ART regimen. In contrast, adjustment for regimen substantially attenuated the decline in first-line treatment modification due to adverse event over time. Significant predictors of first-line treatment modification due to adverse event were earlier period of ART initiation, d4T/NNRTI, AZT/NNRTI or PI-based ART, older age, hepatitis C co-infection and female sex.

ART usage trends presented in this study are mainly a reflection of changes in regional drug availability, ART recommendations and prescriber preferences. In 2006, the WHO guidelines noted the importance of moving away from d4T due to the its long-term toxicity.[5] The WHO 2010 guidelines advised prescribers to employ alternatives wherever possible.[4] On the backdrop of an overall decline in d4T use during the study period and coinciding with the release of the WHO’s recommendations, our results show a substantial drop in d4T use between 2005 and 2006, and again between 2010 and 2011. Unfortunately, the low cost of d4T co-formulations has prevented the drug’s abandonment in Asia and many resource-limited areas.

PI-based ART was more common in Asia prior to 2003 due to the early availability of saquinavir, ritonavir, indinavir, and nelfinavir in higher income countries, while there were few HIV treatment programs in lower-income countries.[15] The subsequent availability of NVP and later EFV, both of which are less costly than PIs and amenable to once-daily dosing, has led to a decline in PI use. Scale-up of ART, particularly NNRTI-based ART, across the region has also contributed to this trend. Increased EFV use in Asia may be ascribed to accumulating anecdotal and scientific evidence of its superior efficacy and safety over NVP [7], [8], the current lack of a once-daily NVP preparation across much of the region, and reductions in price. Nevertheless, NVP use in Asia remains common as it is still cheaper than EFV and is widely available in a variety of co-formulations.

Other studies have evaluated ART usage trends in developed and resource-limited populations.[9]–[12] Similar trends in NRTI and NNRTI use to those found in this study have been reported, although, compared with high-income settings, the decline in d4T use in Asia and Africa has been delayed by several years. At one Spanish center, d4T prescribing was found to have dropped from a peak of 40% of NRTI prescriptions in mid-1999 to <1% by the end of 2006.[11] In contrast, first-line d4T use at sites in Kenya, Uganda and Tanzania began to decline after 2004/5 but remained high by the end of the study period in 2008/9 (68%, 8% and 93% of patients, respectively).[10] Our analysis found d4T use has been declining since 2003 but remained above 5% in 2012/13.

McConnell *et al* (2005) assessed trends in ART usage and long term survival in an observational cohort of HIV infected children and adolescents in the United States between 1989 and 2001.[12] Their results highlighted the increased uptake of triple therapy since 1996 and subsequent improvement in survival for the 1997–2001 group compared with earlier groups. Using similar methods, this study has shown that different patterns of adult first-line ART use in 2003–2006, 2007–2009 and 2010–2013 have significantly contributed to differing rates of toxicity-associated treatment modification across these periods. This bodes well for current and future generations of first-line ART users as fewer treatment modifications equates to less diverse ART use and therefore fewer drug resistance mutations and a greater armamentarium of effective drugs should treatment switch be required. It also suggests retention in care may be enhanced as fewer patients are likely to be discouraged by the onset of adverse events.

A number of advancements have been made to ART in the past decade. For example, the introduction of TDF has provided another safe and efficacious alternative to d4T [16], [17]; the availability of once-daily EFV has provided a more convenient alternative to twice daily NVP that induces less NNRTI resistance [18]; and the development of boosted PIs has allowed smaller, less frequent PI doses that offer improved safety, convenience and efficacy.[19]–[21] Additionally, improvements in general HIV care have been strongly encouraged, particularly in resource-limited settings. These include earlier diagnosis of infected patients, earlier initiation of ART, more extensive patient monitoring, improved retention in treatment programs, and better patient support services.[22] Although we could not capture all influences associated with rates of treatment modification and failure in Asia, this analysis, consistent with similar analyses in non-Asian cohorts,[23], [24] has shown both outcomes are in decline. Importantly, changes to ART prescribing, particularly d4T use, appear to have played an essential role in the encouraging downward trend in treatment modifications due to adverse event.

It was of particular interest in our final multivariate model that females and patients ≥40 years old were at significantly increased risk of treatment modification due to toxicity when compared with males and patients <30 years old, respectively. Many behavioral and societal factors associated with the older female demographic could be considered influential here. However, given the physical, psychological and emotional effects of oestrogen withdrawal during peri-menopause and the potential for these symptoms to persist for several years into the early stages of post-menopause, poorer durability of ART regimens initiated by older women may be associated with the onset of ovarian senescence. In fact, a recent study found HIV-infected, peri-menopausal women experience more severe hot flashes and associated distress when compared with non-HIV-infected peri-menopausal women.[25] There were several limitations to our study. Adverse event reporting was insufficient to delineate treatment modifications related to specific adverse events. The rate of treatment modification is known to increase with improved monitoring [26] hence the rates of failure and modification in earlier years of this analysis may be understated as viral load and CD4 cell count monitoring has expanded in Asia over the past decade. We used observational data from multiple Asian countries with varying income levels and ART accessibility. Therefore, our results may not be representative of the entire Asia region and should not be over interpreted. Poor adherence was uncommon for those that had this data available. Therefore, despite sub-optimal adherence being a well-known predictor of treatment failure, this was not one of our findings. When compared with patients with >95% adherence, those missing adherence data were more likely to fail first-line ART and to experience a treatment modification due to adverse event (Table 2). However, this category represents a mixture of circumstances including: poor adherence; lower frequency of adherence monitoring/support; and earlier study enrolment as adherence data was not collected prospectively prior to 2009 in TAHOD (TASER collected prospective adherence data from its initiation in 2007). Adherence was therefore left out of our final models.

We have described the recent trends in ART use in the TAHOD regional cohort. Changes to the composition of HIV therapies used in Asia over the past decade have contributed to a declining rate of treatment modification due to adverse event but not to reductions in treatment failure.

## Supporting Information

Table S1 **Competing risk models of treatment failure and treatment modification due to adverse event where loss-to-follow-up included as a competing risk (n = 4379).** All models were adjusted for study site. Baseline CD4 cell count and adherence were not significant in univariate analysis for either outcome but are presented out of interest, as is the missing adherence category. Exposure category *Other* includes those exposed to blood products and unknown exposures. A *(d4T/AZT/TDF)/NNRTI* regimen comprises d4T/AZT/TDF + another NRTI + NNRTI. A *PI-based* regimen comprises dual NRTI + PI. *Other* regimen refers to all other ART. ^¤^Included in final treatment failure model; ^◊^Included in final modification due to adverse event model; ^¥^Adjusted for co-variables included in the final model; *Time updated; †p overall for linear trend; ‡p overall for heterogeneity; HR = hazard ratio; ART = antiretroviral therapy; IDU = intravenous drug use; HCV = hepatitis C virus; PI = protease inhibitor; NNRTI = non-nucleoside reverse transcriptase inhibitor; d4T = stavudine; AZT = zidovudine; TDF = tenofovir.
(DOCX)
