# Supplementary material for: Trends in First-Line Antiretroviral Therapy in Asia: Results from the TREAT Asia HIV Observational Database
Source: PLoS One. 2014 Sep 3;9(9):e106525. doi: 10.1371/journal.pone.0106525 (PMC4153611; doi:10.1371/journal.pone.0106525)
Supplement: Table S1 — Competing risk models of treatment failure and treatment modification due to adverse event where loss-to-follow-up included as a competing risk (n = 4379). All models were adjusted for study site. Baseline CD4 cell count and adherence were not significant in univariate analysis for either outcome but are presented out of interest, as is the missing adherence category. Exposure category Other includes those exposed to blood products and unknown exposures. A (d4T/AZT/TDF)/NNRTI regimen comprises d4T/AZT/TDF + another NRTI + NNRTI. A PI-based regimen comprises dual NRTI + PI. Other regimen refers to all other ART. ¤Included in final treatment failure model; ◊Included in final modification due to adverse event model; ¥Adjusted for co-variables included in the final model; *Time updated; †p overall for linear trend; ‡p overall for heterogeneity; HR = hazard ratio; ART = antiretroviral therapy; IDU = intravenous drug use; HCV = hepatitis C virus; PI = protease inhibitor; NNRTI = non-nucleoside reverse transcriptase inhibitor; d4T = stavudine; AZT = zidovudine; TDF = tenofovir. (DOCX) [file pone.0106525.s001.docx]

|  | **Treatment failure** | | | | | | **Modification due to adverse event** | | | | | |
| --- | --- | --- | --- | --- | --- | --- | --- | --- | --- | --- | --- | --- |
| **Co-variables** | **Univariate HR (95%CI)** | **p** | **p overall** | **Multivariate^¥^ HR (95%CI)** | **p** | **p overall** | **Univariate HR (95%CI)** | **p** | **p overall** | **Multivariate^¥^ HR (95%CI)** | **p** | **p overall** |
| **Period of ART initiation^¤◊^** |  |  |  |  |  |  |  |  |  |  |  |  |
| 2003 - 2006 | 1.00 |  |  | 1.00 |  |  | 1.00 |  |  | 1.00 |  |  |
| 2007 - 2010 | 0.64 (0.51 - 0.81) | <0.001 |  | 0.67 (0.53 - 0.84) | **0.001** |  | 0.62 (0.53 - 0.73) | <0.001 |  | 0.79 (0.66 - 0.93) | **0.005** |  |
| 2011 - 2013 | 0.51 (0.33 - 0.79) | 0.003 | <0.001^†^ | 0.54 (0.35 - 0.85) | **0.008** | **0.001^†^** | 0.28 (0.18 - 0.45) | <0.001 | <0.001^†^ | 0.48 (0.30 - 0.76) | **0.002** | **<0.001^†^** |
|  |  |  |  |  |  |  |  |  |  |  |  |  |
| **Initial ART regimen^¤◊^** |  |  |  |  |  |  |  |  |  |  |  |  |
| d4T/NNRTI | 1.55 (1.00 - 2.38) | 0.048 |  | 1.20 (0.77 - 1.87) | 0.418 |  | 13.28 (7.37 - 23.92) | <0.001 |  | 11.25 (6.21 - 20.37) | **<0.001** |  |
| AZT/NNRTI | 1.43 (0.94 - 2.18) | 0.092 |  | 1.24 (0.81 - 1.90) | 0.320 |  | 2.97 (1.63 - 5.42) | <0.001 |  | 2.58 (1.41 - 4.72) | **0.002** |  |
| TDF/NNRTI | 1.00 |  |  | 1.00 |  |  | 1.00 |  |  | 1.00 |  |  |
| PI-based | 1.47 (0.82 - 2.61) | 0.194 |  | 1.28 (0.72 - 2.27) | 0.402 |  | 3.82 (1.98 - 7.34) | <0.001 |  | 3.51 (1.83 - 6.74) | **<0.001** |  |
| Other | 1.90 (1.03 - 3.50) | 0.039 | 0.216^‡^ | 1.79 (0.98 - 3.28) | 0.059 | 0.404^‡^ | 1.61 (0.74 - 3.51) | 0.230 | <0.001^‡^ | 1.62 (0.75 - 3.53) | 0.220 | **<0.001^‡^** |
|  |  |  |  |  |  |  |  |  |  |  |  |  |
| **Age (years)^¤◊^** |  |  |  |  |  |  |  |  |  |  |  |  |
| <30 | 1.00 |  |  | 1.00 |  |  | 1.00 |  |  | 1.00 |  |  |
| 30-39 | 1.22 (0.95 - 1.56) | 0.124 |  | 1.17 (0.91 - 1.51) | 0.212 |  | 1.17 (0.97 - 1.41) | 0.111 |  | 1.12 (0.92 - 1.36) | 0.254 |  |
| 40-49 | 1.25 (0.93 - 1.67) | 0.147 |  | 1.25 (0.92 - 1.68) | 0.153 |  | 1.47 (1.19 - 1.81) | <0.001 |  | 1.56 (1.26 - 1.93) | **<0.001** |  |
| ≥50 | 1.60 (1.13 - 2.26) | 0.008 | 0.015^†^ | 1.61 (1.14 - 2.28) | **0.007** | **0.012^†^** | 1.52 (1.16 - 1.98) | 0.002 | <0.001^†^ | 1.58 (1.20 - 2.07) | **0.001** | **<0.001^†^** |
|  |  |  |  |  |  |  |  |  |  |  |  |  |
| **HCV status^¤◊^** |  |  |  |  |  |  |  |  |  |  |  |  |
| Negative | 1.00 |  |  | 1.00 |  |  | 1.00 |  |  | 1.00 |  |  |
| Positive | 1.50 (1.08 - 2.08) | 0.016 |  | 1.46 (1.04 - 2.03) | **0.026** |  | 1.25 (0.99 - 1.56) | 0.055 |  | 1.30 (1.03 - 1.62) | **0.025** |  |
|  |  |  |  |  |  |  |  |  |  |  |  |  |
| **AIDS prior to ART initiation^¤^** |  |  |  |  |  |  |  |  |  |  |  |  |
| None known | 1.00 |  |  | 1.00 |  |  | 1.00 |  |  | 1.00 |  |  |
| Yes | 1.35 (1.10 - 1.66) | 0.004 |  | 1.28 (1.04 - 1.58) | **0.020** |  | 1.07 (0.92 - 1.23) | 0.390 |  | 0.88 (0.76 - 1.03) | 0.110 |  |
|  |  |  |  |  |  |  |  |  |  |  |  |  |
| **Sex^◊^** |  |  |  |  |  |  |  |  |  |  |  |  |
| Male | 1.00 |  |  | 1.00 |  |  | 1.00 |  |  | 1.00 |  |  |
| Female | 0.80 (0.63 - 1.02) | 0.067 |  | 0.87 (0.68 - 1.10) | 0.246 |  | 1.23 (1.06 - 1.43) | 0.006 |  | 1.28 (1.10 - 1.49) | **0.001** |  |
|  |  |  |  |  |  |  |  |  |  |  |  |  |
| **HIV exposure** |  |  |  |  |  |  |  |  |  |  |  |  |
| Heterosexual | 1.00 |  |  | 1.00 |  |  | 1.00 |  |  | 1.00 |  |  |
| Homosexual | 0.71 (0.51 - 0.98) | 0.038 |  | 0.86 (0.62 - 1.19) | 0.359 |  | 0.65 (0.50 - 0.83) | 0.001 |  | 0.97 (0.73 - 1.29) | 0.854 |  |
| IDU | 1.22 (0.84 - 1.77) | 0.302 |  | 0.96 (0.62 - 1.50) | 0.874 |  | 1.35 (0.99 - 1.83) | 0.055 |  | 1.26 (0.88 - 1.81) | 0.199 |  |
| Other | 0.88 (0.58 - 1.32) | 0.529 | 0.121^‡^ | 0.88 (0.58 - 1.33) | 0.538 | 0.807^‡^ | 0.84 (0.60 - 1.19) | 0.324 | 0.001^‡^ | 0.93 (0.65 - 1.34) | 0.694 | 0.578^‡^ |
|  |  |  |  |  |  |  |  |  |  |  |  |  |
| **Prior mono/dual therapy** |  |  |  |  |  |  |  |  |  |  |  |  |
| None known | 1.00 |  |  | 1.00 |  |  | 1.00 |  |  | 1.00 |  |  |
| Yes | 1.35 (0.97 - 1.88) | 0.071 |  | 1.27 (0.91 - 1.78) | 0.157 |  | 1.16 (0.87 - 1.53) | 0.307 |  | 1.11 (0.84 - 1.47) | 0.463 |  |
|  |  |  |  |  |  |  |  |  |  |  |  |  |
| **Baseline CD4 (cells/mm^3^)** |  |  |  |  |  |  |  |  |  |  |  |  |
| >350 | 1.00 |  |  | 1.00 |  |  | 1.00 |  |  | 1.00 |  |  |
| ≤350 | 0.85 (0.57 - 1.26) | 0.412 |  | 0.79 (0.53 - 1.18) | 0.248 |  | 1.18 (0.81 - 1.74) | 0.389 |  | 0.96 (0.64 - 1.42) | 0.820 |  |
|  |  |  |  |  |  |  |  |  |  |  |  |  |
| **Adherence*** |  |  |  |  |  |  |  |  |  |  |  |  |
| ≥95% | 1.00 |  |  | 1.00 |  |  | 1.00 |  |  | 1.00 |  |  |
| <95% | 1.54 (0.81 - 2.91) | 0.185 |  | 1.58 (0.83 - 3.01) | 0.159 |  | 0.87 (0.52 - 1.45) | 0.594 |  | 0.86 (0.51 - 1.43) | 0.549 |  |
| Missing | 4.53 (3.61 - 5.68) | <0.001 |  | 5.61 (4.32 - 7.29) | <0.001 |  | 2.78 (2.37 - 3.26) | <0.001 |  | 2.16 (1.79 - 2.60) | <0.001 |  |
